# Biomolecular Networks and Human Diseases

**DOI:** 10.1155/2014/363717

**Published:** 2014-06-24

**Authors:** FangXiang Wu, Luonan Chen, Jianxin Wang, Reda Alhajj

**Affiliations:** ^1^Division of Biomedical Engineering, University of Saskatchewan, Saskatoon, SK, Canada S7N 5A9; ^2^Department of Mechanical Engineering, University of Saskatchewan, Saskatoon, SK, Canada S7N 5A9; ^3^Collaborative Research Center for Innovative Mathematical Modelling, Institute of Industrial Science, University of Tokyo, Tokyo 153-8505, Japan; ^4^Key Laboratory of Systems Biology, Shanghai Institutes for Biological Science, Chinese Academy of Sciences, Shanghai 200031, China; ^5^School of Information Science and Engineering, Central South University, Changsha, Hunan 410083, China; ^6^Department of Computer Science, University of Calgary, Calgary, AB, Canada T2N 1N4

Now it is widely acknowledged that genetically caused diseases or disorders (e.g., cancer, AIDS, and obesity) stem from the dysfunction of molecular biological systems, not only their isolated components (e.g., genes, proteins, and metabolites). With advances in high-throughput measurement techniques, large-scale biological data have been and will continuously be produced. Such data contain insightful information for understanding the mechanism of molecular biological systems and have proved useful in diagnosis, treatment, and drug design for genetically caused diseases or disorders. In this special issue, we reported the recent progress in computational approaches that have been developed for analyzing complex networks constructed from high-throughput data and their applications to human diseases.

High-throughput experimental technologies, along with computational predictions, have produced a large amount of protein-protein interaction (PPI) data and thus PPI networks, which makes it possible to understand the role of proteins or genes at the network level. Protein complexes are molecular aggregations of proteins assembled via multiple PPIs. Most proteins are functional only when they are assembled into a protein complex and interact with other proteins in this complex. Therefore, identification of protein complexes from PPI networks has become a key problem for understanding cellular life in postgenomic era. In the paper “*ABC and IFC: modules detection method for PPI network*,” X. Lei et al. proposed a novel clustering model which combined the optimization mechanism of artificial bee colony (ABC) with the fuzzy membership matrix to detect protein complexes from PPI networks. The experimental results on MIPS dataset showed that their proposed ABC-IFC method not only got improved in terms of several commonly used evaluation criteria such as precision, recall, and *P* value but also obtained a better clustering result. Due to the limitation of experiments, there are a substantial number of false positives of the PPIs which can compromise the utility of PPI networks for protein complex detection. To address this issue, a number of data integration and affinity scoring schemes have been devised to get a weighted PPI network. In the paper “*A novel algorithm for detecting protein complexes with the breadth first search*,” X. Tang et al. proposed a novel protein complex mining algorithm ClusterBFS (cluster with breadth first search) to detect the protein complexes from the weighted PPI network. The experimental results showed that ClusterBFS performed significantly better than the other computational approaches in terms of the identification of protein complexes.

Most existing computational methods were applied on static PPI networks to identify the protein complex. However, proteins and their interactions are dynamic in reality. Therefore, identifying dynamic protein complexes is more meaningful and challenging. In their paper “*Identifying dynamic protein complexes based on gene expression profiles and PPI networks*,” M. Li et al. integrated static PPI data and dynamic gene expression profiles to construct dynamic PPI networks and further proposed a novel algorithm, named DPC, to identify dynamic protein complexes. Their proposed algorithm DPC was applied on the data of* Saccharomyces cerevisiae* and the experimental results showed that DPC outperformed CMC, MCL, SPICi, HC-PIN, COACH, and Core-Attachment based on the validation of matching with known complexes and hF-measures. In their paper “*msiDBN: a method of identifying critical proteins in dynamic PPI networks*,” Y. Zhang et al. presented a comprehensive way of modeling the dynamic PPIs and further proposed a novel method, named msiDBN, for modeling a common representation of multiple PPI networks. Experiments were implemented on data of yeast cell cycles. The results of comparison showed that msiDBN had better reconstruction rate and identified more proteins of critical value to yeast cell cycle process. In F. Liu et al.'s paper “*Mining seasonal marine microbial pattern with greedy heuristic clustering and symmetrical nonnegative matrix factorization*,” they developed a novel method called HCsNMF to detect the marine microbial association patterns. The results showed that the four seasonal marine microbial association networks had characters of complex networks.

Cancer is characterized by uncontrolled cell growth as a consequence of activating protooncogenes and/or inactivating tumor suppressor genes. Searching for consistently up- or downregulated genes, proteins, or clusters of them has been the mainstream in identifying potential biomarkers for early cancer diagnosis. However, tumorigenesis as well as cancer progression is a complex and dynamic process. The recent study showed that the characteristics of biomolecular networks for disease cells could be significantly different from those for normal cells. Based on this observation, B. Ling et al. tested the hypothesis that correlations for gene expressions could serve as valid indicators of early cancer development in their paper “*Gene expression correlation for cancer diagnosis: a pilot study.*” Their results showed that strong correlations were observed between genes that are even not on the same pathways during the progression of different cancers, implicating that the correlations for cancer network gene expressions could serve as a supplement to current clinical biomarkers. Cancer starts from normal cells, acquires mutations, and evolves to be malignant cancer cells that are metastatic and/or resistant to therapy. Therefore, identifying driver mutation is important in understanding disease mechanism and future application of custom tailored therapeutic decision. In their paper “*Pathway-driven discovery of rare mutational impact on cancer*,” T. Ahn and T. Park suggested a new approach for discovering rare mutations that have real impact in the context of pathway, which could sensitively capture mutations that change pathway level of mRNA expression. Finding effective anticancer therapies is a major goal of biomedical research. Recently, synthetic lethality (SL) has emerged as a novel anticancer strategy that is promising to be highly selective. In the paper “*Syn-Lethality: an integrative knowledge base of synthetic lethality towards discovery of selective anticancer therapies*,” X. Li et al. presented Syn-Lethality, the first integrative knowledge base of SL that is dedicated to human cancer.

Traditionally, gene sets enrichment analysis of survival related genes is commonly used to reveal the underlying functional mechanisms of complex disease. However, this approach usually produces too many candidate genes and cannot discover detailed signaling transduction cascades, which greatly limits their clinical application such as biomarker development. In the paper “*A network biology approach to discover the molecular biomarker associated with hepatocellular carcinoma*,” L. Zhuang et al. proposed a network biology approach to discover novel biomarkers from multidimensional omics data. Compared with traditional enrichment analysis, this approach can provide concrete and testable hypothesis on functional mechanism. Furthermore, the identified subnetworks from eighty hepatocellular carcinoma (HCC) expression profiling arrays can potentially be used as suitable targets for therapeutic intervention in HCC. In their paper “*Multiple regression analysis of mRNA-miRNA associations in colorectal cancer pathway*,” F. Wang et al. adopted a regression model to identify the significantly associated miRNAs targeting a set of candidate genes frequently involved in colorectal cancer (CRC) pathways. Multiple linear regression analysis was used to construct the model and find the significant mRNA-miRNA associations. The results generated from their study would be helpful in the diagnosis and treatment of CRC. In the paper “*Network of microRNAs-mRNAs interactions in pancreatic cancer*,” E. Naderi et al. constructed the network of miRNA-mRNA interactions for pancreas cancer and illustrated that this network could be used to refine miRNA target predictions for developing new therapeutic approaches.

*FangXiang  Wu* *Luonan  Chen* *Jianxin  Wang* *Reda  Alhajj*

